# Squamous Cell Carcinoma Antigen 2 (SCCA2, SERPINB4): An Emerging Biomarker for Skin Inflammatory Diseases

**DOI:** 10.3390/ijms19041102

**Published:** 2018-04-06

**Authors:** Kenji Izuhara, Yukie Yamaguchi, Shoichiro Ohta, Satoshi Nunomura, Yasuhiro Nanri, Yoshinori Azuma, Noriko Nomura, Yasuhiko Noguchi, Michiko Aihara

**Affiliations:** 1 Division of Medical Biochemistry, Department of Biomolecular Sciences, Saga Medical School, Saga 849-8501, Japan; nunomura@cc.saga-u.ac.jp (S.N.); sp9710@cc.saga-u.ac.jp (Y.N.); 2 Department of Environmental Immuno-Dermatology, Yokohama City University Graduate School of Medicine, Yokohama 236-0004, Japan; yui1783@yokohama-cu.ac.jp (Y.Y.); maihara1@yokohama-cu.ac.jp (M.A.); 3 Department of Medical Technology and Sciences, School of Health Sciences at Fukuoka, International University of Health and Welfare, Okawa 831-8501, Japan; sho.ohta@iuhw.ac.jp; 4 Shino-Test Corporation, Sagamihara 252-0331, Japan; yoshinori.azuma@shino-test.co.jp (Y.A.); noriko.nomura@shino-test.co.jp (N.N.); yan@msd.biglobe.ne.jp (Y.N.)

**Keywords:** SCCA1, SCCA2, SERPIN B3, SERPIN B4, biomarker, psoriasis, atopic dermatitis, IL-22, IL-4, IL-13

## Abstract

Squamous cell carcinoma antigens 1 and 2 (SCCA1 and 2, SERPIN B3 and B4), members of the ovalbumin serpin (ov-serpin)/clade B serpin family, were originally discovered as tumor-specific antigens and are used as tumor markers for various kinds of squamous cell carcinomas. Recently, our understanding of the underlying mechanisms of how SCCA1/2 enhance tumor growth has greatly increased. Moreover, it has been shown that SCCA1/2 are involved in the pathogenesis of several inflammatory diseases: asthma, psoriasis, and atopic dermatitis (AD). IL-22 and IL-17, signature cytokines of type 17 inflammation, as well as IL-4 and IL-13, signature cytokines of type 2 inflammation, both of which are positively correlated with the pathogenesis of psoriasis and allergic diseases, respectively, can induce expression of SCCA1/2 in airway epithelial cells and/or keratinocytes, leading to high expression of SCCA1/2 in these diseases. Based on these findings, several trials have been performed to examine the potential of applying SCCA1/2 to biomarkers for these diseases. The findings show that SCCA2 is useful to aid diagnosis, estimate clinical severity and disease type, and assess responses to treatment in psoriasis and AD. These results suggest that SCCA2 has emerged as a novel biomarker for skin inflammatory diseases.

## 1. Introduction

Squamous cell carcinoma antigens 1 and 2 (SCCA1 and 2, SERPIN B3 and B4) are members of the ovalbumin serpin (ov-serpin)/clade B serpin family [1] and were originally discovered as tumor-specific antigens in the uterine cervix [2]. SCCA1 and SCCA2 are highly homologous proteins, 91% identical at the amino acid level, and probably evolving from a common ancestor gene [1]. However, they have different target proteases. SCCA1 inhibits papain-like cysteine proteases such as papain, cathepsin-S, -K, and -L, whereas SCCA2 inhibits both serine and cysteine proteases such as cathepsin-G, mast cell chymase, *Dermatophagoides pteronyssinus* 1 (*Der p* 1) and *Dermatophagoides farinae* 1 (*Der f* 1). SCCA molecules are used as tumor markers for various kind of squamous cell carcinomas—esophagus, lung, head and neck, anal canal, and uterine cervix—reflecting tumor stage, tumor size, stromal invasion, lymph-vascular space status, and lymph node status [3]. Since we published a review article on the ov-serpin/clade B serpin family including SCCA1 and SCCA2 [1], the understanding of the functions of SCCA molecules has expanded, and the usefulness of SCCA molecules, particularly SCCA2, as biomarkers for skin inflammatory diseases such as psoriasis and atopic dermatitis (AD) has emerged. In this article, we discuss these recent developments.

## 2. Development in Understanding the Functions of SCCA Molecules

**Figure 1:**
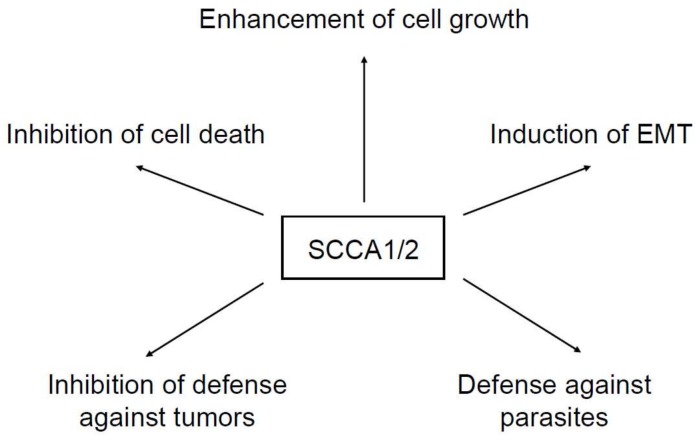
Functions of squamous cell carcinoma antigens (SCCA) molecules. The functions of SCCA molecules are summarized as (1) inhibition of cell death, (2) enhancement of cell growth, (3) induction of epithelial–mesenchymal transition (EMT), (4) inhibition of defense against tumors, and (5) defense against parasites.

In view of the high expression of SCCA molecules in various kinds of tumor cells, a number of studies have been performed to clarify the roles of these molecules in oncogenesis. It has been established that SCCA molecules enhance tumor growth in vivo; overexpression of SCCA1 in lung tumor cells enhanced the tumor size in nude mice, whereas silencing of SCCA1/2 by antisense decreased it [4,5]. Extensive research to understand how SCCA1/2 enhance tumor growth has been carried out. The underlying mechanisms have been summarized as (1) inhibition of cell death, (2) enhancement of cell growth, (3) induction of epithelial–mesenchymal transition (EMT), and (4) inhibition of defense against tumors (Figure 1). These actions can be applied to non-malignant as well as malignant cells. Moreover, independent of the actions on malignant cells, we have proposed a fifth mechanism: defense against parasites as a novel role of SCCA in vivo.

### 2.1. Inhibition of Cell Death

Suminami et al. previously demonstrated that overexpression of SCCA1 in several kinds of tumor cells protected against apoptosis induced by an anti-cancer drug (7-ethyl-10-hydroxycamptothecin), by TNF-α, and by IL-2–activated natural killer (NK) cells, whereas silencing SCCA1 increased sensitivity to apoptosis induced by an anti-cancer drug (etoposide) and by TNF-α [4]. They showed that overexpression of SCCA1 inhibited the activity of caspase-3, a cysteine-protease critical for the apoptosis cascade, which could be an underlying mechanism of anti-apoptosis activity by SCCA1. However, it was unclear whether SCCA1 directly inhibited the protease activity of either caspase-3 or its upstream proteases. The protective activities of both SCCA1 and SCCA2 against apoptosis were also observed in irradiated tumor cells, in which inactivation of p38 MAPK followed by inhibition of caspase-3 and -9 was involved, although overexpression of SCCA1/2 in tumor cells did not change the proliferation [6]. Katagiri et al. expanded the anti-apoptotic activities of SCCA into keratinocytes [7]. They observed high expression of SCCA in UV-irradiated epidermis and showed that UV-induced apoptosis or UV-induced skin damage was decreased in SCCA1/2-overexpressed fibroblasts or in SCCA1-transgenic mice, respectively, whereas knockdown of SCCA1 in keratinocytes increased the sensitivity to UV-induced apoptosis. Moreover, they showed that SCCA1 inhibited JNK activity by binding to JNK and translocating to the nucleus, which was compatible with their finding that SCCA1 was strongly stained in nuclei as well as cytoplasm in some UV-irradiated epidermis. This could be a mechanism of anti-apoptosis by SCCA1 in keratinocytes exposed to UV. Ciscato et al. proposed another mechanism of anti-apoptosis by SCCA1 via mitochondria [8]. They showed that SCCA1 was translocated into mitochondria during treatment of tumor cells with anti-cancer drugs. SCCA1 then interacted with Complex I followed by inhibiting generation of reactive oxygen species (ROS) and opening of the mitochondrial permeability transition pore (PTP). In contrast to these reports showing SCCA1/2 inhibiting cell apoptosis, Ullman et al. proposed that SCCA1 enhanced endoplasmic reticulum (ER) stress-induced cell apoptosis by causing subsequent cleavage and activation of caspase-8, whereas SCCA1 protected against cell necrosis induced by lysosomal injury [9]. 

### 2.2. Enhancement of Cell Growth

It has been recently demonstrated that SCCA1/2 are correlated with the oncogenic activities of mutated Ras and Myc, both of which are well known oncoproteins. Catanzaro et al. demonstrated that oncogenic Ras induced SCCA1/2 via the ETS family transcriptional factor, PEA3 [10]. SCCA1/2 promoted NF-κB signaling followed by enhanced production of cytokines such as IL-6, IL-8, CXCL1, G-CSF, and GM-CSF by blocking protein turnover and inducing unfolded protein response (UPR), leading to tumor growth. Turato et al. showed SCCA1 up-regulation of Myc and its translocation to the nucleus. SCCA1 upregulated expression of Myc via Yes-associated protein (Yap) and inhibited calpain—a cysteine protease cleaving Myc—which would be an underlying mechanism of up-regulation of Myc by SCCA1 [11].

### 2.3. Induction of EMT

EMT is an important event for oncogenesis by enhancing invasion and metastasis of tumor cells [12]. EMT is a process that is well accepted in vitro, whereas evidence of its existence in vivo are not so clear. Quarta et al. demonstrated that SCCA1 plays an important role in EMT [13]. Exogenous addition of SCCA1 to tumor cells caused morphological changes of cells, decrease of desmosomal junctions and widening of intercellular spaces, and reduction of E-cadherin and increase of β-catenin, all of which correspond to features of EMT. Overexpression of SCCA1 induced cell scattering, migration, and invasiveness, which in turn were inhibited by the addition of neutralizing antibodies (Abs) against SCCA1. Interestingly, the action of SCCA1 in inducing EMT was independent of its anti-protease activity.

### 2.4. Inhibition of the Immune System against Tumors

Cytotoxic T cells and natural killer (NK) cells play a pivotal role in eliminating tumor cells [14]. Suminami et al. demonstrated that SCCA1, but not SCCA2, inhibited MCP-1–induced chemotaxis of NK cells dependent on its anti-protease activity [5]. It is known that cytotoxic T cells and NK cells secrete five different granzymes (GzmA, GzmB, GzmH, GzmK, and GzmH), serine proteases, and that granzyme-mediated cell death is the major pathway to kill virus-infected cells or tumor cells [14]. On the other hand, it is known that SERPINB9 inhibits GzmM [1]. A study by de Koning et al. showed that SCCA2 inhibited protease activity of GzmB, another granzyme, dependent of its anti-protease activity [15]. These findings suggest that SCCA1 and/or SCCA2 may play a role in preventing the immune system from defending against tumors by inhibiting chemotaxis or cell toxicity of NK cells.

### 2.5. Defense System against Parasites

Many protozoan and helminth parasites generate abundant Clan CA family C1 cysteine proteases, which are orthologues of cathepsin L [16]. These cysteine proteases play critical roles in differentiation of parasites, infection to the hosts, and immunomodulation of the hosts. In addition, it is well known that type 2 immunity is important for the defense mechanism against parasites [17]. As we will describe below, we found that IL-4 and IL-13, signature cytokines of type 2 immunity, induce expression of SCCA1 and SCCA2 in epithelial cells [18]. Therefore, we reasoned that SCCA1 and SCCA2 would be secreted from epithelial cells stimulated by IL-4 or IL-13 to create a defense system against parasite infection. We found that SCCA1, but not SCCA2, potently inhibited various cysteine proteases derived from parasites *Leishmania mexicana*, *Trypanosoma cruzi*, *Trypanosoma brucei rhodesience*, and *Fasciola hepatica*. We proposed the possibility that SCCA molecules are equipped to be a defense mechanism against parasites [19].

Thus, diverse functions of SCCA molecules have been reported. The locations of SCCA molecules are also various: cytoplasm, lysosome, mitochondria, nucleus, and extracellular space [20]. Target proteases of SCCA have been sometimes specified and sometimes not. Moreover, SCCA1 and SCCA2 that have different target proteases sometimes show the same biological activities exerting their functions, independently of their anti-protease activities. We need further studies to define the molecular mechanisms of the functions of SCCA1 and SCCA2.

### 2.6. Developments in Measuring Separately SCCA1 and SCCA2

SCCA1 and SCCA2 are broadly co-expressed in normal tissues, including the epithelium of tongue, tonsil, esophagus, uterine cervix, vagina, the conducting airways, Hassall’s corpuscles of the thymus, and some areas of the skin [21]. However, since SCCA1 and SCCA2 have different target proteases, it is of particular interest to examine how expression of SCCA1 and SCCA2 differs in various pathophysiological conditions and how we can apply the difference to clinical medicine. Although several studies concerning the differing expressions of SCCA1 and SCCA2 at the mRNA level were reported [22], no system or method to separate measurement of SCCA1 and SCCA2 at the protein level had been established. However, it would be difficult to generate monoclonal antibodies (mAbs) specifically recognizing either SCCA1 or SCCA2 because these two proteins are highly homologous, 91% identical at the amino acid level [1].

**Figure 2:**
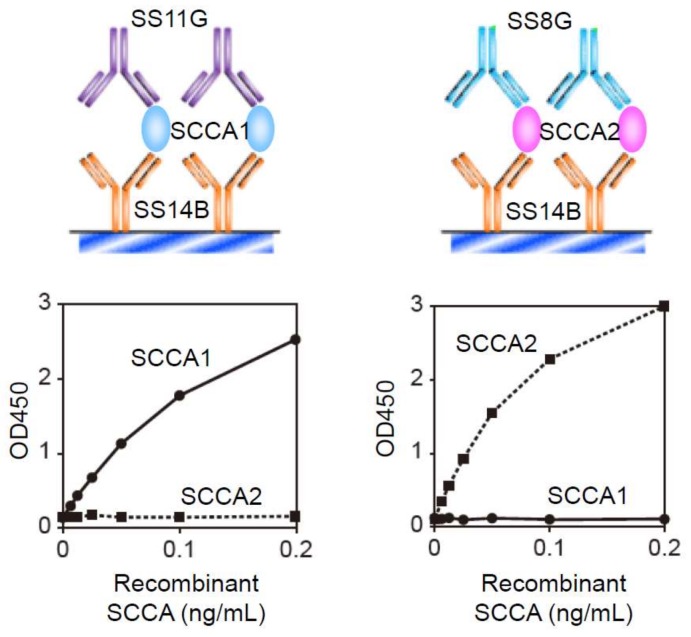
The ELISA systems to recognize specifically SCCA1 and SCCA2 (modified from [24]). The ELISA systems using SS14B as the coating Ab and SS11G and SS8G for SCCA1 or SCCA2 as the primary Abs, respectively, recognize specifically SCCA1 and SCCA2.

Çataltepe et al. succeeded in establishing an ELISA system to recognize specifically SCCA1 and SCCA2 [23]. In this system, they used anti-SCCA rabbit serum as the coating Ab and anti-SCCA1 Ab (clone 8H11) or anti-SCCA2 Ab (clone 10C12) as the primary mAb. These systems clearly distinguished SCCA1 and SCCA2 and gave 0.17 ng/mL and 0.19 ng/mL as the detection limits of SCCA1 and SCCA2, respectively; 12.1% and 12% for the inter-assay coefficients of variation (CV) of SCCA1 and SCCA2, respectively; and 9.9% and 8.8% for the intra-assay of CV of SCCA1 and SCCA2, respectively. We then succeeded in establishing another detection system to measure separately SCCA1 and SCCA2 (Figure 2) [24]. In our system, we used SS14B, an anti-SCCA mAb recognizing both SCCA1 and SCCA2 as the coating Ab, and SS11G and SS8G, mAbs recognizing SCCA1 or SCCA2, respectively, as the primary Abs. The detection limits of our system using labeling with biotin for SCCA1 and SCCA2 were 1.5 pg/mL and 0.8 pg/mL, respectively, and the intra-assay CVs for SCCA1 and SCCA2 were 0.81–5.2% and 0.64–5.32% [24]. Moreover, the detection limit of our system using labeling with peroxidase for SCCA2 was 3 pg/mL, and the inter- and intra-assay CVs for SCCA2 were 3.3–8.7% and 0.8–5.2% [25]. These showed the superior ability of our system.

## 3. SCCA Molecules in Asthma

Asthma is a chronic airway inflammatory disease characterized by respiratory symptoms such as wheezing, shortness of breath, chest tightness, and coughing, together with expiratory airflow limitation. Although asthma is a heterogeneous disease, it is thought that 50–80% of asthma patients are type 2 inflammation-dominant [26,27,28] and that type 2 inflammation generates asthma-specific phenotypes such as goblet cell hyperplasia, eosinophil-dominant infiltration of inflammatory cells, thickened basement membrane, and hyperplasia or hypertrophy of smooth muscle cells [29]. IL-4 and IL-13 are signature type 2 cytokines playing critical roles in the pathogenesis of asthma. Moreover, it has been shown, using model mice, that the actions of IL-13 on airway epithelial cells are critical for generating asthma-like phenotypes [30,31,32,33].

### 3.1. Involvement of SCCA1/2 in Asthma as Downstream Molecules of IL-4/IL-13

**Figure 3:**
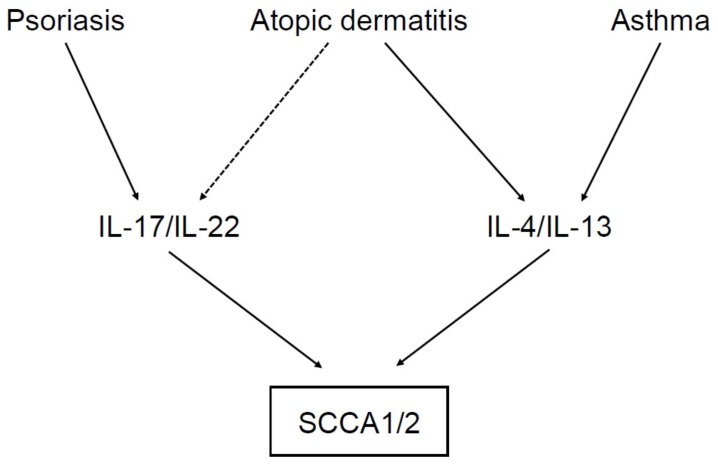
Induction of SCCA1 and SCCA2 in inflammatory diseases. IL-17/IL-22 and IL-4/IL-13, signature cytokines of type 17 and type 2 inflammation, respectively, can induce SCCA1 and SCCA2 in keratinocytes or airway epithelial cells. IL-17/IL-22 are highly expressed in psoriasis patients, and IL-4/IL-13 are highly expressed in atopic dermatitis (AD) and asthma patients. In some AD patients, IL-17/IL-22 are also expressed.

To clarify the underlying mechanism of how IL-4 and/or IL-13 generates asthma-like phenotypes by acting on airway epithelial cells, we studied IL-4/IL-13–inducible genes in those cells using a DNA microarray and found that SCCA1 and SCCA2 are downstream molecules of IL-4/IL-13 (Figure 3) [18]. The ability of IL-4 or IL-13 to induce expression of SCCA1 and SCCA2 in airway epithelial cells was also confirmed by other studies [34,35]. Karaaslan et al. demonstrated that one polymorphism on the SCCA1 gene, GAGG/AGCTT (rs116864116), was associated with eosinophil count, skin test positivity, and IgE, but not with asthma incidence, and that the variant type had higher binding activities with GATA2 and GATA3 stimulated by IL-4 and IL-13 [36].

### 3.2. SCCA in Mouse Models of Asthma

It is known that in rodents, orthologs of human ov-serpins are well conserved, expanding the repertoire of several orthologs including SERPINB3 [1]. With regard to SERPINB3-related molecules, there exist four members: Serpinb3a, b3b, b3c, and b3d. The homologies of Serpinb3a with SCCA1 and SCCA2 are 60% and 61%, respectively. We found that Serpinb3a and Serpinb3b are functional as serpin molecules, whereas Serpinb3c is non-functional [37]. The function of Serpinb3d is unknown. The functions of Serpinb3a and Serpinb3b were overlapping; Serpinb3a can inhibit cathepsin L, cathepsin G, *Der p 1*, and mast cell chymase, whereas Serpin b3b can inhibit cathepsin L and cathepsin G, but not *Der p 1* or mast cell chymase. This contrasts with the characteristics of SCCA1 and SCCA2; these two serpin molecules complement target proteases. The distributions of Serpinb3a and Serpinb3b were also different; Serpinb3a was ubiquitously expressed, whereas Serpinb3b was expressed only in keratinocytes, as far as we surveyed. Moreover, neither IL-4 nor IL-13 could induce expression of Serpinb3a and Serpinb3b, and these molecules were constitutively expressed in keratinocytes. These results suggest that the mouse orthologs of SCCA1 and SCCA2 would exhibit pathophysiological roles similar to, but to some extent different from, human orthologs. 

Uteroglobin/clara cell 10 kDa protein (CC10) is a steroid-inducible, multifunctional, secreted protein with potent anti-inflammatory and immunomodulatory protein [38]. Uteroglobin/CC10 deficiency caused asthma-like airway inflammation, together with eosinophil-dominance and high expression of type 2 cytokines, including IL-4 and IL-13 [39,40]. Ray et al. showed that Serpinb3a was highly expressed in airway epithelial cells in uteroglobin/CC10-deficient mice and that allergen exposure enhanced expression of Serpinb3a compared to either non-exposed uteroglobin/CC10-deficient mice or to exposed wild-type mice [34]. These results suggest that expression of Serpinb3a is upregulated in allergic inflammation to the same extent as SCCA1 and SCCA2, although it is unclear whether IL-4 or IL-13 directly induces Serpinb3a expression. 

Using Serpinb3a-deficient mice, Sivaprasad et al. directly examined the roles of Serpinb3a in the pathogenesis of airway allergic inflammation [35]. Deficiency of Serpinb3a did not change allergen sensitization, but significantly downregulated mite- or IL-13-induced airway hyperresponsiveness, mucus production, and infiltration of inflammatory cells such as eosinophils. The SAM-pointed domain-containing ETS transcriptional factor (SPDEF) and forkhead box transcription factor A3 (FOXA3), are both important for goblet cell hyperplasia. SPDEF is a downstream molecule of IL-13 [41,42]. Expression of both molecules was decreased in Serpinb3a-deficient mice. These findings, taken together, show that Serpinb3a plays an important role in airway allergic inflammation. The mechanism causing it remains unclear, but it would reflect the roles of SCCA1 and SCCA2, which are highly expressed in asthma patients. 

### 3.3. Usefulness of SCCA as a Biomarker for Asthma

In 1998, the National Institutes of Health Biomarkers Definitions Working Group defined a biomarker as a characteristic that is objectively measured and evaluated as an indicator of normal biologic processes, pathogenic processes, or pharmacologic responses to a therapeutic intervention [43]. The types of biomarkers include pharamacodynamic, predictive, diagnostic, and prognostic ones [44]. In the case of asthma, physiological tests such as lung functions and airway hypersensitivity, eosinophil in sputum or in nasal discharge, fraction of exhaled nitric oxide (FeNO), and total or specific IgE are currently used as biomarkers [44]. Moreover, periostin has emerged as a novel biomarker for asthma, reflecting type 2 inflammation and tissue remodeling in asthma patients [45,46]. However, the repertoire of biomarkers for asthma, particularly those in serum, is limited. Although the SCCA genes do not have signal sequences, we found that SCCA molecules could be secreted from cells via an ER/Golgi-independent pathway [19]. Therefore, we explored the possibility that SCCA molecules would be biomarkers for treating asthma.

We first performed a pilot study to compare serum SCCA levels in asthmatic children and in control children [18]. Since most childhood asthma is atopic [47], we would expect that SCCA levels would be upregulated in asthmatic children. In this study, we used the SCCA ELISA assay (IMx, Abbott, Tokyo, Japan), which detects only SCCA1. SCCA levels in asthmatic children were only slightly higher than those of control subjects (median: 2.1 ng/mL, interquartile range: 1.4–3.3 ng/mL vs. median: 1.1 ng/mL, interquartile range: 0.6–1.9 ng/mL, *p* < 0.005). When we compared the SCCA levels in the exacerbation stage and in the convalescence stage in a small population, SCCA levels tended to be higher in the exacerbation stage (median: 2.4 ng/mL, interquartile range: 1.7–3.8 ng/mL) than in the convalescence stage (median: 1.4 ng/mL, interquartile range: 0.9–1.7 ng/mL, *p* < 0.005), although statistical significance was not reached (*p* = 0.09). Nishi et al. expanded this study, examining a larger population [48]. When they looked at SCCA levels in the recovery stage of asthmatic children and in control children, the levels were comparable (*n* = 35, 4.65 ± 0.57 ng/mL vs. *n* = 21, 5.71 ± 0.86 ng/mL). In contrast, SCCA levels in the acute phase were significantly higher than those of the recovery phase (3.09 ± 2.03 ng/mL vs. 1.47 ± 0.64 ng/mL, *n* = 35, *p* < 0.0001). Neither disease severity, serum IgE, regular use of inhaled corticosteroids, a history of intravenous hydrocortisone injection during an acute attack, nor the presence of AD was positively correlated with SCCA levels in either the acute or the recovery phase. Serum IL-13 levels were positively correlated with SCCA levels in the recovery phase, but not in the acute phase, suggesting that SCCA, a downstream molecule of IL-4/IL-13, indicates type 2 inflammation. Another study compared both SCCA1 and SCCA2 levels in asthmatic children; SCCA1 levels were slightly higher in asthmatic children than in control children (median: 0.88 ng/mL, interquartile range: 0.57–1.28 ng/mL, vs. median: 0.55 ng/mL, interquartile range: 0.41–0.83 ng/mL, *p* = 0.011), whereas there was no difference in SCCA2 in these groups. Moreover, Nakamura et al. have recently published analyses of SCCA1 and SCCA2 levels in infants and children younger than 36 months hospitalized with RSV-induced bronchitis [49]. They showed that SCCA1 levels were higher in the acute phase than in the recovery phase, both in patients with modified asthma predictive index (mAPI, 3.51 ± 2.34 ng/mL vs. 1.29 ± 0.66 ng/mL, *n* = 14, *p* = 0.002) and without mAPI (2.96 ± 1.67 ng/mL vs. 1.47 ± 0.85 ng/mL, *n* = 22, *p* = 0.001). SCCA2 levels also showed the same tendency; SCCA2 levels were higher in the acute phase than in the recovery phase both in those with mAPI (1.43 ± 0.80 ng/mL vs. 0.95 ± 0.76 ng/mL, *n* = 14, *p* = 0.019) and without mAPI (1.60 ± 0.31 ng/mL vs. 1.19 ± 1.13 ng/mL, *n* = 22, *p* = 0.002). mAPI is the index used to predict the probability of future asthma onset in pediatric patients. These results suggest that SCCA1/2 are not appropriate as asthma biomarkers, but instead reflect the destruction of airway epithelial cells. 

## 4. SCCA Molecules in Psoriasis

Psoriasis is a chronic relapsing inflammatory disease characterized by erythematous papules and plaques with white thick scales secondary to excessive growth of skin epithelial cells [50,51,52]. It has long been recognized that psoriasis is a T cell-mediated autoimmune disease. Recently, researchers investigating the pathogenesis of psoriasis have postulated that the TNF/IL-23/type 17 inflammation axis is central to it [50,52,53]. A great deal of effort has gone into finding biomarkers for psoriasis; however, no biomarker has yet been translated into routine clinical practice [54,55]. Topical agents (corticosteroids, vitamin D analogues), phototherapy, methotrexate, and cyclosporine were until recently the major treatments for psoriasis [52,56]. Based on our current understanding of the immunopathogenesis of psoriasis, biologics have emerged that target TNF, IL-12/IL-23p40 subunit, IL-23p19 subunit, IL-17A, and the IL-17 receptor α chain (IL-17RA) [50,52,53].

### 4.1. Involvement of SCCA1/2 in Psoriasis as Downstream Molecules of IL-22 and IL-17

Several comprehensive analyses of gene expression profiles in psoriasis have shown that both SCCA1 and SCCA2 are highly expressed in the inflamed skin of psoriasis patients. An analysis of skin biopsies from psoriasis patients and normal volunteers using an mRNA differential display method showed that four genes including SCCA1 were highly upregulated in the patients’ skin [57]. Two analyses to compare the gene expression profiles in lesional and non-lesional skin of psoriasis patients demonstrated that expression of both SCCA1 and SCCA2 was upregulated in inflamed skin [58,59]. It is of note that SCCA2 and SCCA1 were ranked as the most highly and the fifth most highly upregulated genes, respectively [59]. Guttman-Yassky et al. compared gene expression profiles in psoriasis and AD patients, finding that both SCCA1 and SCCA2 were upregulated in skin from both psoriasis and AD patients, compared with normal skin, and that expression was higher in psoriasis patients than in AD patients [60]. Moreover, a meta-analysis based on three microarray data studies to compare lesional and adjacent non-lesional skin of psoriasis patients showed that SCCA2 and SCCA1 were ranked as the most highly and the 11th most highly upregulated genes [61]. These results suggest that both SCCA1 and SCCA2 are reproducibly and significantly upregulated molecules in lesional skin of psoriasis patients. 

**Figure 4:**
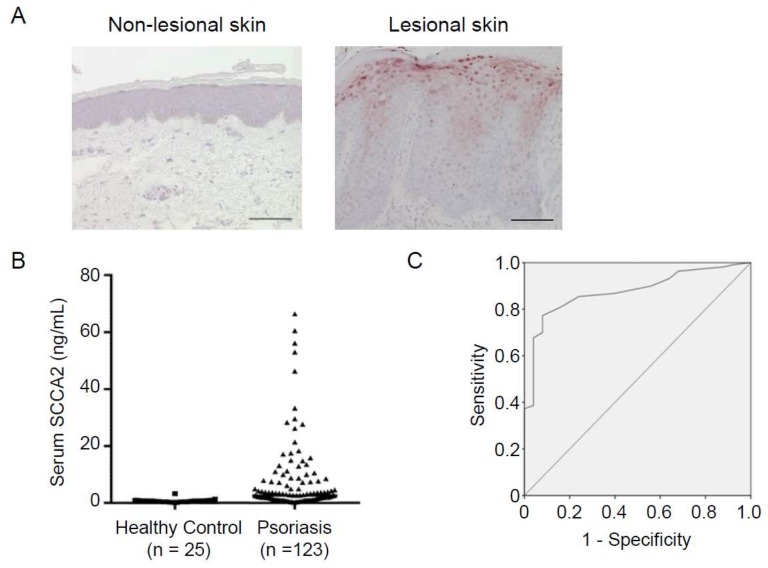
Involvement of SCCA in psoriasis (modified from [63]). (**A**) Immunohistochemical analysis of SCCA2 expression in a psoriasis patient. Non-lesional (left) and lesional (right) skin. Scale bar = 100 µm. (**B**) Serum SCCA2 levels in healthy controls and overall psoriasis patients. (**C**) Receiver operating characteristic (ROC) analysis comparing psoriasis patients and healthy controls.

Histochemical analyses of SCCA in psoriatic skin showed that SCCA was highly expressed through the suprabasal to the granular layer of epidermis, associated with severity (Figure 4) [57,62,63]. Interestingly, it was shown that SCCA was observed in the nuclei of the granular layer cells and in elongated rete ridges in addition to around the plasma membrane or intercellular space [62,63]. Taking into account that SCCA in nuclei plays a role in anti-apoptotic action, periostin highly expressed in nuclei may protect keratinocytes from apoptosis, contributing to hyperkeratosis in psoriasis. It has been reported that SCCA can be an autoantigen in psoriasis, although its pathological role or significance remains obscure [64]. 

We examined the ability of each cytokine correlated with psoriasis to enhance expression of SCCA2. We found that IL-22 had a potent ability and IL-17A had a less ability to do so and that in keratinocytes, the combination of IL-22 and IL-17A significantly enhanced expression of SCCA2 (Figure 3) [63]. Induction of both SCCA1 and SCCA2 by IL-22 in keratinocytes was also observed in another study [59]. These results demonstrate that SCCA1/2 are downstream molecules of IL-22 and IL-17 and explain why these molecules are highly expressed in psoriasis.

### 4.2. Usefulness of SCCA as a Biomarker for Psoriasis

Based on the knowledge that SCCA molecules are highly expressed in psoriatic skin and that SCCAs are downstream molecules of IL-22/IL-17 tightly correlated with pathogenesis of psoriasis, we explored the usefulness of SCCA2 as a biomarker for psoriasis [63]. We chose SCCA2 because it had been reported that SCCA2 is dominantly expressed in lesional skin of psoriasis patients compared with SCCA1 [59,61], and because we had already established an ELISA kit specifically recognizing SCCA2 [24]. Serum SCCA levels in overall psoriasis patients were significantly higher than in healthy volunteers (Figure 4, *n* = 123, median: 2.7 ng/mL, interquartile range: 1.25–7.75 ng/mL, vs. *n* = 25, median: 0.70 ng/mL, interquartile range: 0.40–0.80 ng/mL, *p* < 0.0001), but comparable with AD patients (*n* = 34, median: 3.38 ng/mL, interquartile range: 2.02–6.30 ng/mL). All three types of psoriasis—psoriasis vulgaris, psoriasis arthritis, and pustular psoriasis—showed higher SCCA levels (*n* = 96, median: 2.65 ng/mL, interquartile range: 1.175–7.75 ng/mL, *p* < 0.0001, *n* = 16, median: 2.1 ng/mL, interquartile range: 0.825–3.55 ng/mL, *p* < 0.01, and *n* = 11, median: 4.9 ng/mL, interquartile range: 1.55–10.6 ng/mL, *p* < 0.0001, respectively) than healthy controls, with no statistically significant difference among these three groups. The area under the receiver operating characteristic (ROC) curve (AUC) between psoriasis patients and healthy volunteers was 0.877, giving us high specificity (77.3%) and sensitivity (92%) with the cut-off value of 1.05 ng/mL. Serum SCCA2 levels were significantly correlated with severity of psoriasis, using Psoriasis Area Severity Index (PASI) scores (*r* = 0.68, *p* < 0.0001). Moreover, SCCA2 levels were well correlated with serum IL-22 (*p* < 0.05), but not IL-17A or IL-36γ, consistent with the finding that IL-22 is the most dominant inducer of SCCA2. Based on these findings, SCCA2 has emerged as a useful biomarker indicating the severity of psoriasis.

## 5. SCCA Molecules in AD

AD is a chronic or relapsing, inflammatory skin disease characterized by persistent pruritus and recurrent eczema [65]. It is widely accepted that type 2 and Th22 inflammation are dominant in the pathogenesis of AD [65]. However, AD pathogenesis is thought to be heterogeneous [66]; both type 2 and non-type 2 AD exist [67]. Moreover, ethnic differences in the pathogenesis of AD have been proposed: AD in European American patients is more type 2 dominant, and AD in certain East Asian patients (Japanese + Korean) is mixed with type 2 and type 17 inflammation [68]. Topical corticosteroids and immunosuppresive drugs are used as basic treatments. Recently, dupilumab, an antagonist against the IL-4 receptor α chain (IL-4Rα) important for the signals of both IL-4 and IL-13, signature cytokines of type 2 inflammation, has been launched as the first molecularly targeted drug for treating AD patients [69]. It had been claimed that there was no reliable biomarker for AD [70]. However, a systematic review has recently shown that thymus and activation-regulated chemokine (TARC)/CCL17, a chemokine involved in type 2 inflammation, is the most reliable biomarker indicating the disease severity of AD [71]. Moreover, we have showed that periostin is a novel useful biomarker for AD to help in diagnosis, assess clinical severity and disease type, and reflect response to treatment [72].

### 5.1. Involvement of SCCA1/2 in AD as Downstream Molecules of IL-4/IL-13

**Figure 5:**
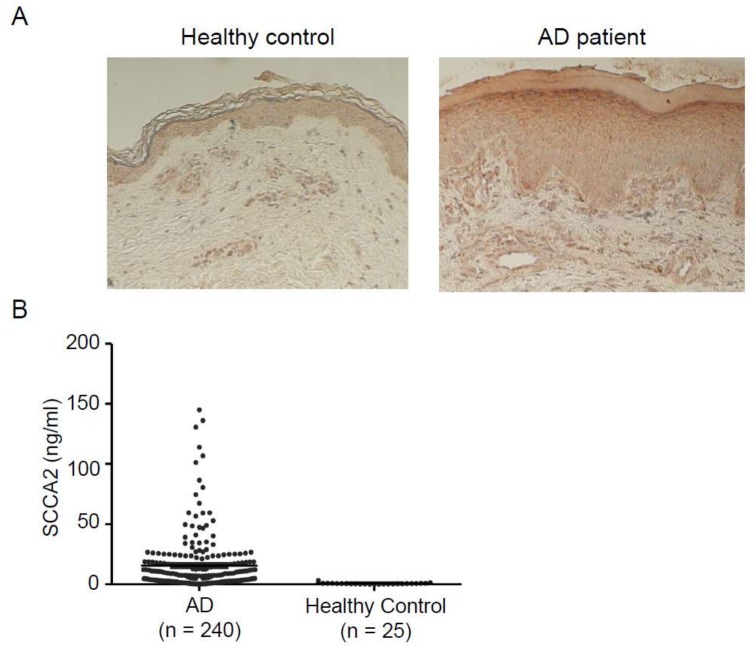
Involvement of SCCA in adult AD (modified from [76]). (**A**) Immunohistochemical analysis of SCCA2 expression in an adult AD patient. Skin from a healthy control (left) and an adult AD patient (right). (**B**) Serum SCCA2 levels in healthy controls and overall AD patients.

Several comprehensive analyses to examine the gene expression profile of AD patients showed that expression of both SCCA1 and SCCA2 was upregulated in lesional AD skin compared with skin derived from normal subjects [60,73]. Moreover, Mitsuishi et al. showed that expression of both SCCA1 and SCCA2 was higher in lesional than in either non-lesional or normal skin [74]. Using histochemical analysis, they showed that SCCA was highly deposited in the cytoplasm of keratinocytes particularly, in the spinous layer and the intercellular bridges. Yamane et al. also showed that expression of SCCA2 at protein levels was upregulated in lesional skin of AD patients compared with non-lesional or normal skin and was positively correlated with total IgE levels [75]. We also confirmed the strong expression of SCCA2 in the spinous to the granular layer and that SCCA2 was located in the cytoplasm of keratinocytes (Figure 5) [76]. These results suggest that in lesional skin of AD patients, the expression of both SCCA1 and SCCA2 is induced mainly by IL-4 and IL-13 and in some patients, possibly by IL-22 and IL-17 (Figure 3). 

### 5.2. SCCA in a Mouse Model of AD

Using Serpinb3a-deficient mice, Sivaprasad et al. examined the role of SCCA in the pathogenesis of AD [77]. When Serpinb3a-deficinet mice were exposed to *Aspergillus fumigatus*, these mice showed decreases in transepidermal water loss, skin lesion score (redness, skin thickening, and excoriations), and epidermal thickness, compared with wild-type mice. Moreover, upregulated expression of various pro-inflammatory genes including S100A8 was impaired in *Aspergillus fumigatus*-exposed Serpinb3a-deficinet mice. Knockdown of both SCCA1/2 in human keratinocytes also downregulated S1008 expression. These findings using Serpinb3a-deficient mice support the pro-inflammatory effects of SCCA molecules in the pathogenesis of AD as well as of asthma.

### 5.3. Usefulness of SCCA as a Biomarker for AD

In the initial studies to analyze SCCA levels in AD patients, these levels were found to be upregulated, tightly correlated with clinical severity [74,78]. The correlation of SCCA levels with laboratory parameters reflecting type 2 inflammation was controversial: one paper showed good correlations of SCCA levels with eosinophil numbers, LDH, and total IgE [74], whereas another paper did not show a statistically significant correlation with either eosinophil numbers or IgE [78].

**Figure 6:**
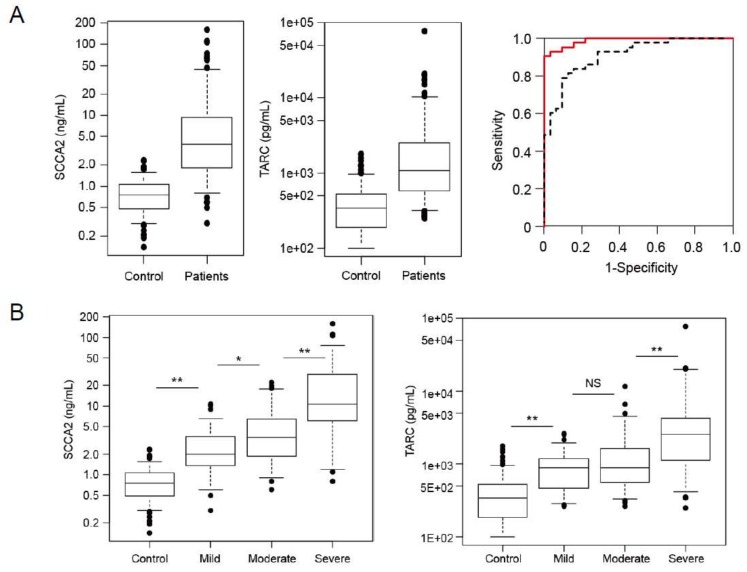
Usefulness of SCCA2 as a biomarker for pediatric AD patients (modified from [25]). (**A**) Serum SCCA2 and thymus and activation-regulated chemokine (TARC) levels in control subjects and AD children and ROC curve analysis of SCCA2 (red solid line) and TARC in all AD children. (**B**) Association of Objective Scoring Atopic Dermatitis (O-SCORAD) with SCCA2 and TARC in the AD children. It is of note that the area under the curve (AUC) for SCCA2 is higher than that of TARC in panel A and that serum SCCA2 levels show better correlation with O-SCORAD than do serum TARC levels. *p < 0.01, ** *p* < 0.001.

After we established ELISA systems to distinguish SCCA1 and SCCA2, we explored the usefulness of measuring SCCA2 in both adult and pediatric AD patients because in vitro analyses had shown that SCCA2, compared with SCCA1, is dominantly expressed by IL-4 or IL-13 in keratinocytes [24,25,76]. We examined serum SCCA2 levels in adult AD patients (Figure 5) [76]. These levels went up according to clinical severity and had different characteristics depending on clinical types: They showed high levels in the erythroderma type and in the presence of lichenification, whereas they showed low levels in the prurigo type. Serum SCCA2 levels were well associated with other type 2 biomarkers—TARC, blood eosinophils, and total IgE—and with LDH. Serum SCCA2 levels went down after appropriate treatment. A pilot study for pediatric AD patients (aged 1.3 ± 0.82 years) with food allergies showed high levels of both SCCA1 and SCCA2, with higher levels of SCCA2 correlated with clinical severity [24]. We have recently reported the results of a multicenter analysis to compare SCCA2 and TARC in pediatric AD patients [25]. Serum levels of both SCCA2 and TARC were high in AD children (Figure 6); however, ROC analyses showed that SCCA2 corresponded more closely to diagnosis of AD than did TARC in any age group (AUC; SCCA2:0.929, TARC: 0.871 in all subjects). Serum levels of both SCCA2 and TARC went up in accordance with clinical severity as estimated by Objective Scoring Atopic Dermatitis (O-SCORAD); however, SCCA2 was better able to distinguish clinical severities in AD children. The abilities of SCCA2 and TARC to assess the effects of topical corticosteroid treatment were almost the same. These results suggest that SCCA2 is a more reliable biomarker than TARC to correspond to diagnosis of AD and to estimate its clinical severity in children. Since periostin—another novel biomarker for allergic diseases—is found at high levels in infants and children [46,79], SCC2 has a superiority as a biomarker for them, compared with periostin. IL-22 and IL-17 may be involved in elevated SCCA2 levels, but not TARC levels, in some AD patients, to which the superiority of SCCA2 compared with TARC would be ascribed. It is of note that although three cut-off levels for TARC are used, depending on age [80], only one cut-off value for SCCA2 irrespective of age, 1.6 ng/mL, provided an acceptable result in that it was highly correlated with the diagnosis of AD (79.5% of sensitivity and 95% of specificity across all ages). That value would provide clinicians the convenience of using SCCA2 compared with TARC.

## 6. Perspectives

In the past decade, our understanding of the functions of SCCA molecules has grown. With it, the usefulness of the SCCA molecules, particularly SCCA2, for treating inflammatory skin diseases psoriasis and AD has emerged. However, several questions remain. Although the underlying mechanism of how SCCA molecules enhance tumor growth has been well clarified, it is still obscure how SCCA molecules enhance inflammation in vivo, as shown in several mouse models. Moreover, many molecularly targeted drugs for psoriasis and AD—TNF, IL-12/IL-23p40 subunit, IL-23p19 subunit, IL-17A, IL-17RA, and IL-4Rα—have been developed. Diagnostics to estimate the efficacy of these drugs, so-called “companion diagnostics,” are needed. Moreover, diagnostics to monitor the efficacy of these drugs and to access the time points to stop these drugs are still to be developed. It will be of particular interest to examine whether SCCA2 has such abilities. Further research aiming at these points should be undertaken.

## References

[1] Izuhara K., Ohta S., Kanaji S., Shiraishi H., Arima K. (2008). Recent progress in understanding the diversity of the human ov-serpin/clade B serpin family. Cell. Mol. Life Sci..

[2] Kato H., Torigoe T. (1977). Radioimmunoassay for tumor antigen of human cervical squamous cell carcinoma. Cancer.

[3] Gadducci A., Tana R., Cosio S., Genazzani A.R. (2008). The serum assay of tumour markers in the prognostic evaluation, treatment monitoring and follow-up of patients with cervical cancer: A review of the literature. Crit. Rev. Oncol. Hematol..

[4] Suminami Y., Nagashima S., Vujanovic N.L., Hirabayashi K., Kato H., Whiteside T.L. (2000). Inhibition of apoptosis in human tumour cells by the tumour-associated serpin, SCC antigen-1. Br. J. Cancer.

[5] Suminami Y., Nagashima S., Murakami A., Nawata S., Gondo T., Hirakawa H., Numa F., Silverman G.A., Kato H. (2001). Suppression of a squamous cell carcinoma (SCC)-related serpin, SCC antigen, inhibits tumor growth with increased intratumor infiltration of natural killer cells. Cancer Res..

[6] Murakami A., Suminami Y., Hirakawa H., Nawata S., Numa F., Kato H. (2001). Squamous cell carcinoma antigen suppresses radiation-induced cell death. Br. J. Cancer.

[7] Katagiri C., Nakanishi J., Kadoya K., Hibino T. (2006). Serpin squamous cell carcinoma antigen inhibits UV-induced apoptosis via suppression of c-JUN NH2-terminal kinase. J. Cell Biol..

[8] Ciscato F., Sciacovelli M., Villano G., Turato C., Bernardi P., Rasola A., Pontisso P. (2014). SERPINB3 protects from oxidative damage by chemotherapeutics through inhibition of mitochondrial respiratory complex I. Oncotarget.

[9] Ullman E., Pan J.A., Zong W.X. (2011). Squamous cell carcinoma antigen 1 promotes caspase-8-mediated apoptosis in response to endoplasmic reticulum stress while inhibiting necrosis induced by lysosomal injury. Mol. Cell. Biol..

[10] Catanzaro J.M., Sheshadri N., Pan J.A., Sun Y., Shi C., Li J., Powers R.S., Crawford H.C., Zong W.X. (2014). Oncogenic Ras induces inflammatory cytokine production by upregulating the squamous cell carcinoma antigens SerpinB3/B4. Nat. Commun..

[11] Turato C., Cannito S., Simonato D., Villano G., Morello E., Terrin L., Quarta S., Biasiolo A., Ruvoletto M., Martini A. (2015). SerpinB3 and Yap interplay increases Myc oncogenic activity. Sci. Rep..

[12] Steinestel K., Eder S., Schrader A.J., Steinestel J. (2014). Clinical significance of epithelial-mesenchymal transition. Clin. Transl. Med..

[13] Quarta S., Vidalino L., Turato C., Ruvoletto M., Calabrese F., Valente M., Cannito S., Fassina G., Parola M., Gatta A. (2010). SERPINB3 induces epithelial-mesenchymal transition. J. Pathol..

[14] Cullen S.P., Martin S.J. (2008). Mechanisms of granule-dependent killing. Cell Death Differ..

[15] De Koning P.J., Kummer J.A., de Poot S.A., Quadir R., Broekhuizen R., McGettrick A.F., Higgins W.J., Devreese B., Worrall D.M., Bovenschen N. (2011). Intracellular serine protease inhibitor SERPINB4 inhibits granzyme M-induced cell death. PLoS ONE.

[16] Sajid M., McKerrow J.H. (2002). Cysteine proteases of parasitic organisms. Mol. Biochem. Parasitol..

[17] Allen J.E., Sutherland T.E. (2014). Host protective roles of type 2 immunity: Parasite killing and tissue repair, flip sides of the same coin. Semin. Immunol..

[18] Yuyama N., Davies D.E., Akaiwa M., Matsui K., Hamasaki Y., Suminami Y., Yoshida N.L., Maeda M., Pandit A., Lordan J.L. (2002). Analysis of novel disease-related genes in bronchial asthma. Cytokine.

[19] Kanaji S., Tanaka Y., Sakata Y., Takeshita K., Arima K., Ohta S., Hansell E.J., Caffrey C., Mottram J.C., Lowther J. (2007). Squamous Cell Carcinoma Antigen 1 is an Inhibitor of Parasite-Derived Cysteine Proteases. FEBS Lett..

[20] Sun Y., Sheshadri N., Zong W.X. (2017). SERPINB3 and B4: From biochemistry to biology. Semin. Cell Dev. Biol..

[21] Cataltepe S., Gornstein E.R., Schick C., Kamachi Y., Chatson K., Fries J., Silverman G.A., Upton M.P. (2000). Co-expression of the squamous cell carcinoma antigens 1 and 2 in normal adult human tissues and squamous cell carcinomas. J. Histochem. Cytochem..

[22] Stenman J., Hedstrom J., Grenman R., Leivo I., Finne P., Palotie A., Orpana A. (2001). Relative Levels of SCCA2 and SCCA1 mRNA in Primary Tumors Predicts Recurrent Disease in Squamous Cell Cancer of the Head and Neck. Int. J. Cancer.

[23] Cataltepe S., Schick C., Luke C.J., Pak S.C., Goldfarb D., Chen P., Tanasiyevic M.J., Posner M.R., Silverman G.A. (2000). Development of Specific Monoclonal Antibodies and a Sensitive Discriminatory Immunoassay for the Circulating Tumor Markers SCCA1 and SCCA2. Clin. Chim. Acta.

[24] Ohta S., Shibata R., Nakao Y., Azuma Y., Taniguchi K., Arima K., Suzuki S., Shiraishi H., Iwasaka T., Izuhara K. (2012). The usefulness of combined measurements of squamous cell carcinoma antigens 1 and 2 in diagnosing atopic dermatitis. Ann. Clin. Biochem..

[25] Nagao M., Inagaki S., Kawano T., Azuma Y., Nomura N., Noguchi Y., Ohta S., Kawaguchi A., Odajima H., Ohya Y. (2018). SCCA2 is a reliable biomarker for evaluating pediatric atopic dermatitis. J. Allergy Clin. Immunol..

[26] Woodruff P.G., Modrek B., Choy D.F., Jia G., Abbas A.R., Ellwanger A., Koth L.L., Arron J.R., Fahy J.V. (2009). T-helper type 2-driven inflammation defines major subphenotypes of asthma. Am. J. Respir. Crit. Care Med..

[27] Jia G., Erickson R.W., Choy D.F., Mosesova S., Wu L.C., Solberg O.D., Shikotra A., Carter R., Audusseau S., Hamid Q. (2012). Periostin is a Systemic Biomarker of Eosinophilic Airway Inflammation in Asthmatic Patients. J. Allergy Clin. Immunol..

[28] Matsusaka M., Fukunaga K., Kabata H., Izuhara K., Asano K., Betsuyaku T. (2018). Subphenotypes of type 2 severe asthma in adults. J. Allergy Clin. Immunol. Pract..

[29] Fahy J.V. (2015). Type 2 inflammation in asthma—Present in most, absent in many. Nat. Rev. Immunol..

[30] Grünig G., Warnock M., Wakil A.E., Venkayya R., Brombacher F., Rennick D.M., Sheppard D., Mohrs M., Donaldson D.D., Locksley R.M., Corry D.B. (1998). Requirement for IL-13 independently of IL-4 in experimental asthma. Science.

[31] Wills-Karp M., Luyimbazi J., Xu X., Schofield B., Neben T.Y., Karp C.L., Donaldson D.D. (1998). Interleukin-13: Central mediator of allergic asthma. Science.

[32] Zhu Z., Homer R.J., Wang Z., Chen Q., Geba G.P., Wang J., Zhang Y., Elias J.A. (1999). Pulmonary expression of interleukin-13 causes inflammation, mucus hypersecretion, subepithelial fibrosis, physiologic abnormalities, and eotaxin production. J. Clin. Investig..

[33] Kuperman D.A., Huang X., Koth L.L., Chang G.H., Dolganov G.M., Zhu Z., Elias J.A., Sheppard D., Erle D.J. (2002). Direct effects of interleukin-13 on epithelial cells cause airway hyperreactivity and mucus overproduction in asthma. Nat. Med..

[34] Ray R., Choi M., Zhang Z., Silverman G.A., Askew D., Mukherjee A.B. (2005). Uteroglobin suppresses SCCA gene expression associated with allergic asthma. J. Biol. Chem..

[35] Sivaprasad U., Askew D.J., Ericksen M.B., Gibson A.M., Stier M.T., Brandt E.B., Bass S.A., Daines M.O., Chakir J., Stringer K.F. (2011). A nonredundant role for mouse Serpinb3a in the induction of mucus production in asthma. J. Allergy Clin. Immunol..

[36] Karaaslan C., Birben E., Keskin O., Sahiner U., Sackesen C., Kalayci O. (2013). The role of SCCA1 in asthma related physiological events in the airway epithelium and the effect of promoter variants on asthma and gene function. Respir. Med..

[37] Sakata Y., Arima K., Takeshita K., Takai T., Aoki S., Ogawa H., Sugihara H., Fujimoto K., Izuhara K. (2004). Characterization of novel squamous cell carcinoma antigen-related molecules in mice. Biochem. Biophys. Res. Commun..

[38] Mukherjee A.B., Kundu G.C., Mantile-Selvaggi G., Yuan C.J., Mandal A.K., Chattopadhyay S., Zheng F., Pattabiraman N., Zhang Z. (1999). Uteroglobin: A novel cytokine?. Cell. Mol. Life Sci..

[39] Mandal A.K., Zhang Z., Ray R., Choi M.S., Chowdhury B., Pattabiraman N., Mukherjee A.B. (2004). Uteroglobin represses allergen-induced inflammatory response by blocking PGD2 receptor-mediated functions. J. Exp. Med..

[40] Chen L.C., Zhang Z., Myers A.C., Huang S.K. (2001). Cutting edge: Altered pulmonary eosinophilic inflammation in mice deficient for Clara cell secretory 10-kDa protein. J. Immunol..

[41] Park K.S., Korfhagen T.R., Bruno M.D., Kitzmiller J.A., Wan H., Wert S.E., Khurana Hershey G.K., Chen G., Whitsett J.A. (2007). SPDEF regulates goblet cell hyperplasia in the airway epithelium. J. Clin. Investig..

[42] Chen G., Korfhagen T.R., Xu Y., Kitzmiller J., Wert S.E., Maeda Y., Gregorieff A., Clevers H., Whitsett J.A. (2009). SPDEF is required for mouse pulmonary goblet cell differentiation and regulates a network of genes associated with mucus production. J. Clin. Investig..

[43] Atkinson A.J., Colburn W.A., DeGruttola V.G., DeMets D.L., Downing G.J., Hoth D.F., Oates J.A., Peck C.C., Schooley R.T., Spilker B.A. (2001). Biomarkers and surrogate endpoints: Preferred definitions and conceptual framework. Clin. Pharmacol. Ther..

[44] Yancey S.W., Keene O.N., Albers F.C., Ortega H., Bates S., Bleecker E.R., Pavord I. (2017). Biomarkers for severe eosinophilic asthma. J. Allergy Clin. Immunol..

[45] Izuhara K., Matsumoto H., Ohta S., Ono J., Arima K., Ogawa M. (2015). Recent developments regarding periostin in bronchial asthma. Allergol. Int..

[46] Izuhara K., Nunomura S., Nanri Y., Ogawa M., Ono J., Mitamura Y., Yoshihara T. (2017). Periostin in inflammation and allergy. Cell. Mol. Life Sci..

[47] Fainardi V., Saglani S. (2015). The need to differentiate between adults and children when treating severe asthma. Expert Rev. Respir. Med..

[48] Nishi N., Miyazaki M., Tsuji K., Hitomi T., Muro E., Zaitsu M., Yamamoto S., Inada S., Kobayashi I., Ichimaru T. (2005). Squamous cell carcinoma-related antigen in children with acute asthma. Ann. Allergy Asthma Immunol..

[49] Nakamura H., Akashi K., Watanabe M., Ohta S., Ono J., Azuma Y., Ogasawara N., Yamamoto K., Shimizu N., Tsutsumi H. (2017). Up-regulation of serum periostin and squamous cell carcinoma antigen levels in infants with acute bronchitis due to respiratory syncytial virus. Allergol. Int..

[50] Woo Y.R., Cho D.H., Park H.J. (2017). Molecular Mechanisms and Management of a cutaneous inflammatory disorder: Psoriasis. Int. J. Mol. Sci..

[51] Boutet M.A., Nerviani A., Gallo Afflitto G., Pitzalis C. (2018). Role of the IL-23/IL-17 axis in psoriasis and psoriatic arthritis: The clinical importance of its divergence in skin and joints. Int. J. Mol. Sci..

[52] Greb J.E., Goldminz A.M., Elder J.T., Lebwohl M.G., Gladman D.D., Wu J.J., Mehta N.N., Finlay A.Y., Gottlieb A.B. (2016). Psoriasis. Nat. Rev. Dis. Primers.

[53] Conrad C., Gilliet M. (2018). Psoriasis: From pathogenesis to targeted therapies. Clin. Rev. Allergy Immunol..

[54] Jiang S., Hinchliffe T.E., Wu T. (2015). Biomarkers of an autoimmune skin disease—Psoriasis. Genom. Proteom. Bioinform..

[55] Villanova F., Di Meglio P., Nestle F.O. (2013). Biomarkers in psoriasis and psoriatic arthritis. Ann. Rheum. Dis..

[56] Ritchlin C.T., Krueger J.G. (2016). New therapies for psoriasis and psoriatic arthritis. Curr. Opin. Rheumatol..

[57] Rivas M.V., Jarvis E.D., Morisaki S., Carbonaro H., Gottlieb A.B., Krueger J.G. (1997). Identification of aberrantly regulated genes in diseased skin using the cDNA differential display technique. J. Investig. Dermatol..

[58] Suarez-Farinas M., Lowes M.A., Zaba L.C., Krueger J.G. (2010). Evaluation of the psoriasis transcriptome across different studies by gene set enrichment analysis (GSEA). PLoS ONE.

[59] Gudjonsson J.E., Ding J., Johnston A., Tejasvi T., Guzman A.M., Nair R.P., Voorhees J.J., Abecasis G.R., Elder J.T. (2010). Assessment of the psoriatic transcriptome in a large sample: Additional regulated genes and comparisons with in vitro models. J. Investig. Dermatol..

[60] Guttman-Yassky E., Suarez-Farinas M., Chiricozzi A., Nograles K.E., Shemer A., Fuentes-Duculan J., Cardinale I., Lin P., Bergman R., Bowcock A.M. (2009). Broad defects in epidermal cornification in atopic dermatitis identified through genomic analysis. J. Allergy Clin. Immunol..

[61] Tian S., Krueger J.G., Li K., Jabbari A., Brodmerkel C., Lowes M.A., Suarez-Farinas M. (2012). Meta-analysis derived (MAD) transcriptome of psoriasis defines the “core” pathogenesis of disease. PLoS ONE.

[62] Takeda A., Higuchi D., Takahashi T., Ogo M., Baciu P., Goetinck P.F., Hibino T. (2002). Overexpression of serpin squamous cell carcinoma antigens in psoriatic skin. J. Investig. Dermatol..

[63] Watanabe Y., Yamaguchi Y., Komitsu N., Ohta S., Azuma Y., Izuhara K., Aihara M. (2016). Elevation of serum squamous cell carcinoma antigen 2 in patients with psoriasis: Associations with disease severity and response to the treatment. Br. J. Dermatol..

[64] El-Rachkidy R.G., Young H.S., Griffiths C.E., Camp R.D. (2008). Humoral autoimmune responses to the squamous cell carcinoma antigen protein family in psoriasis. J. Investig. Dermatol..

[65] Furue M., Chiba T., Tsuji G., Ulzii D., Kido-Nakahara M., Nakahara T., Kadono T. (2017). Atopic dermatitis: Immune deviation, barrier dysfunction, IgE autoreactivity and new therapies. Allergol. Int..

[66] Bieber T. (2012). Atopic dermatitis 2.0: From the clinical phenotype to the molecular taxonomy and stratified medicine. Allergy.

[67] Thijs J.L., Strickland I., Bruijnzeel-Koomen C., Nierkens S., Giovannone B., Csomor E., Sellman B.R., Mustelin T., Sleeman M.A., de Bruin-Weller M.S. (2017). Moving toward endotypes in atopic dermatitis: Identification of patient clusters based on serum biomarker analysis. J. Allergy Clin. Immunol..

[68] Noda S., Suarez-Farinas M., Ungar B., Kim S.J., de Guzman Strong C., Xu H., Peng X., Estrada Y.D., Nakajima S., Honda T. (2015). The Asian atopic dermatitis phenotype combines features of atopic dermatitis and psoriasis with increased TH17 polarization. J. Allergy Clin. Immunol..

[69] Mennini M., Dahdah L., Fiocchi A. (2017). Two Phase 3 Trials of Dupilumab Versus Placebo in Atopic Dermatitis. N. Engl. J. Med..

[70] Eichenfield L.F., Tom W.L., Chamlin S.L., Feldman S.R., Hanifin J.M., Simpson E.L., Berger T.G., Bergman J.N., Cohen D.E., Cooper K.D. (2014). Guidelines of care for the management of atopic dermatitis: Section 1. Diagnosis and assessment of atopic dermatitis. J. Am. Acad. Dermatol..

[71] Thijs J., Krastev T., Weidinger S., Buckens C.F., de Bruin-Weller M., Bruijnzeel-Koomen C., Flohr C., Hijnen D. (2015). Biomarkers for atopic dermatitis: A systematic review and meta-analysis. Curr. Opin. Allergy Clin. Immunol..

[72] Kou K., Okawa T., Yamaguchi Y., Ono J., Inoue Y., Kohno M., Matsukura S., Kambara T., Ohta S., Izuhara K. (2014). Periostin levels correlate with disease severity and chronicity in patients with atopic dermatitis. Br. J. Dermatol..

[73] Lu Z.R., Park T.H., Lee E.S., Kim K.J., Park D., Kim B.C., Cho S.W., Bhak J., Park Y.D., Zou F. (2009). Dysregulated genes of extrinsic type of atopic dermatitis: 34K microarray and interactomic analyses. J. Dermatol. Sci..

[74] Mitsuishi K., Nakamura T., Sakata Y., Yuyama N., Arima K., Sugita Y., Suto H., Izuhara K., Ogawa H. (2005). The squamous cell carcinoma antigens as relevant biomarkers of atopic dermatitis. Clin. Exp. Allergy.

[75] Yamane Y., Moriyama K., Yasuda C., Miyata S., Aihara M., Ikezawa Z., Miyazaki K. (2009). New horny layer marker proteins for evaluating skin condition in atopic dermatitis. Int. Arch. Allergy Immunol..

[76] Okawa T., Yamaguchi Y., Kou K., Ono J., Azuma Y., Komitsu N., Inoue Y., Kohno M., Matsukura S., Kambara T. (2018). Serum levels of squamous cell carcinoma antigens 1 and 2 reflect disease severity and clinical type of atopic dermatitis in adult patients. Allergol. Int..

[77] Sivaprasad U., Kinker K.G., Ericksen M.B., Lindsey M., Gibson A.M., Bass S.A., Hershey N.S., Deng J., Medvedovic M., Khurana Hershey G.K. (2015). SERPINB3/B4 contributes to early inflammation and barrier dysfunction in an experimental murine model of atopic dermatitis. J. Investig. Dermatol..

[78] Kawashima H., Nishimata S., Kashiwagi Y., Numabe H., Sasamoto M., Iwatsubo H., Takekuma K., Hoshika A. (2000). Squamous cell carcinoma-related antigen in children with atopic dermatitis. Pediatr. Int..

[79] Inoue Y., Izuhara K., Ohta S., Ono J., Shimojo N. (2015). No increase in the serum periostin level is detected in elementary school-age children with allergic diseases. Allergol. Int..

[80] Fujisawa T., Nagao M., Hiraguchi Y., Katsumata H., Nishimori H., Iguchi K., Kato Y., Higashiura M., Ogawauchi I., Tamaki K. (2009). Serum measurement of thymus and activation-regulated chemokine/CCL17 in children with atopic dermatitis: Elevated normal levels in infancy and age-specific analysis in atopic dermatitis. Pediatr. Allergy Immunol..

